# Beyond Early Initiation: Predictors of Successful Early Enteral Nutrition Advancement in Critically Ill Patients

**DOI:** 10.3390/nu18121977

**Published:** 2026-06-18

**Authors:** Jungwon Cho, Ahreum Shin, Chami Im

**Affiliations:** 1 Department of Pharmacy, Seoul National University Bundang Hospital, Gumi-ro 173, Bundang-gu, Seongnam-si 13620, Gyeonggi-do, Republic of Korea; xcully@snubh.org; 2 Department of Clinical Nutrition, Seoul National University Bundang Hospital, Gumi-ro 173, Bundang-gu, Seongnam-si 13620, Gyeonggi-do, Republic of Korea; 20232@snubh.org; 3 Department of Surgery, Seoul National University Bundang Hospital, Gumi-ro 173, Bundang-gu, Seongnam-si 13620, Gyeonggi-do, Republic of Korea; 4 Department of Surgery, College of Medicine, Seoul National University Yeongeon Campus, 103 Daehak-ro, Jongno-gu, Seoul 03080, Republic of Korea

**Keywords:** enteral nutrition, early enteral nutrition, enteral nutrition advancement, intensive care unit, lactate, APACHE II, nutrition support

## Abstract

**Background/Objectives**: Early enteral nutrition (EN) initiation and progressive EN advancement are critical components of nutritional care in critically ill patients; however, not all patients achieve successful early EN advancement in real-world intensive care unit (ICU) settings. We investigated clinical predictors of early EN initiation and successful early EN advancement at ICU admission in a retrospective cohort study at a single tertiary academic medical center in South Korea. **Methods**: A total of 2112 critically ill adults receiving EN between January 2020 and December 2024 were included. Successful early EN advancement was defined as EN initiation within 48 h of ICU admission, followed by progressive advancement without any reduction or discontinuation during the subsequent seven days. Using a two-stage multivariable logistic regression approach, we identified predictors of each outcome. **Results**: Among the total cohort, 722 patients (34.2%) achieved early EN initiation, of whom 449 (62.2%) subsequently achieved successful early EN advancement, representing 21.3% of the total cohort. Male sex (adjusted odds ratio [aOR] 0.87, 95% CI 0.78–0.96), higher admission lactate (aOR 0.85, 95% CI 0.74–0.96), prior surgery (aOR 0.81, 95% CI 0.70–0.93), and higher APACHE II score (aOR 0.88, 95% CI 0.79–0.99) were identified as significant negative predictors (all *p* < 0.05). Admission-time variables (male sex, elevated lactate, prior surgery, and higher APACHE II scores) effectively identify patients at risk of early EN failure. **Conclusions**: Reflecting distinct predictor profiles between ICU types, the preliminary nomogram can guide tailored nutritional strategies, although prospective external validation remains essential before clinical implementation.

## 1. Introduction

Critically ill patients admitted to the intensive care unit (ICU) are at substantial risk of malnutrition, which can worsen preexisting nutritional deficits [1,2,3,4] and contribute to higher mortality and prolonged hospital stays [5,6,7,8]. Given these consequences, timely and appropriate nutrition therapy is considered a cornerstone of ICU management, and growing evidence supports the integration of structured nutrition delivery plans into critical care practice [9,10,11,12,13].

Among available nutritional modalities, enteral nutrition (EN) is preferred in critically ill patients for its ability to preserve gastrointestinal mucosal integrity, modulate immune function, and reduce infectious complications [14,15]. The European Society for Clinical Nutrition and Metabolism (ESPEN) recommends initiating EN within 48 h of ICU admission, given its clinical advantages over delayed EN or parenteral nutrition (PN) [14]. Beyond the timing of initiation, the rate of EN advancement following initiation has also been shown to independently influence clinical outcomes [16,17,18]. Our previous study demonstrated that rapid EN advancement was associated with reduced in-hospital mortality and shortened hospital stay in critically ill patients, with the benefit being particularly pronounced in those who received early EN initiation within 48 h of ICU admission [19]. These findings suggest that achieving both early initiation and rapid advancement of EN—herein defined as “successful early EN advancement”—represents an important clinical target in ICU nutrition management.

However, successful early EN advancement remains difficult to achieve in real-world ICU settings. Multiple patient-related and clinical factors may impede EN initiation or interrupt its progression, including hemodynamic instability, gastrointestinal intolerance, frequent procedural interruptions, and competing clinical priorities [20,21]. While previous studies have primarily focused on either the timing of EN initiation or caloric adequacy as separate endpoints, few have examined the determinants of the combined outcome of early initiation followed by uninterrupted advancement. Understanding which patients are likely to fail this combined goal—and identifying these patients early at the time of ICU admission—could allow clinicians to implement preemptive strategies such as closer monitoring, protocol-driven advancement, or earlier consideration of supplemental PN. Despite the clinical importance of this combined outcome, its predictors remain poorly characterized. Most existing prediction models for EN outcomes have focused on feeding intolerance or caloric deficit rather than the sequential process of initiation followed by sustained advancement [22,23].

Therefore, this study aimed to identify the predictors of early EN initiation and successful early EN advancement in critically ill patients, using clinical variables available at the time of ICU admission. Furthermore, we developed a clinical prediction nomogram to provide a practical bedside tool for estimating the likelihood of achieving successful early EN advancement.

## 2. Materials and Methods

### 2.1. Study Design and Patients

This retrospective cohort study included 2112 critically ill adults who received EN at Seoul National University Bundang Hospital (SNUBH) over a 60-month period, from January 2020 to December 2024. Adult patients were eligible for inclusion if they were admitted to the ICU and received EN during their stay.

SNUBH is a 1224-bed academic teaching hospital in South Korea, providing comprehensive critical care and advanced life support for patients with high-acuity illnesses. SNUBH houses multiple specialized ICUs—including medical, neurological, surgical, and cardiovascular units—each managed by dedicated intensive care-led teams and equipped for complex multi-organ support.

Nutritional management in ICUs is guided by a multidisciplinary nutrition support team (NST) comprising a physician, nurse, dietitian, and pharmacist. At SNUBH, EN initiation and advancement are guided by a standardized protocol under the supervision of a multidisciplinary NST. EN is generally initiated at a low rate and advanced incrementally toward the target caloric goal, with adjustments made based on gastrointestinal tolerance and hemodynamic stability. While individual clinician judgment may influence day-to-day decisions, the overarching framework is consistent across all ICU units [19,24]. Following a collaborative care approach previously described, the NST identifies patients’ nutritional status, provides individualized therapy, and monitors for adverse reactions.

### 2.2. Data Collection

Data were extracted from the SNUBH electronic database. Patient demographics, including age, sex, body mass index (BMI), serum lactate level at ICU admission, operation history, department of admission, type of ICU, and Acute Physiology and Chronic Health Evaluation II (APACHE II) score at ICU admission, were collected.

To ensure the robustness of the predictive model, we applied the following exclusion criteria: (1) procedural ICU admission; (2) ICU readmission on the same day of discharge; and (3) missing discharge date (hospitalized status at the time of data collection).

Patients were classified into three ICU types according to the clinical specialty of the primary admitting department, rather than the physical location of the ICU unit: neurological ICU (neurology), medical ICU (internal medicine and emergency medicine), and surgical ICU (general surgery, neurosurgery, thoracic surgery, and other surgical specialties). All surgeries performed under general anesthesia were included in the surgery variable; these patients were admitted to the surgical ICU based on their admitting department.

### 2.3. Outcomes and Definition

This study evaluated clinical predictors of “early EN initiation” and “successful early EN advancement” at ICU admission. The primary outcome was successful early EN advancement, defined as EN initiation within 48 h of ICU admission followed by progressive advancement of EN without any interruption, reduction, or discontinuation during the subsequent seven days. Patients who started EN therapy but did not meet this definition were classified as the comparator group. This 7-day advancement period was defined in accordance with ESPEN guidelines recommending progressive EN advancement to caloric targets within the first 3–7 days of ICU admission [14] and is consistent with the timeframe used in our previous work demonstrating the clinical benefit of rapid EN advancement [18].

As an exploratory outcome, early EN initiation was analyzed separately, defined as EN administration within 48 h of ICU admission regardless of subsequent advancement.

### 2.4. Statistical Analysis

Patient characteristics are presented as the mean ± standard deviation for continuous variables and as frequencies (percentages) for categorical variables. Differences between groups were assessed using Student’s *t*-test for continuous variables and the chi-square test for categorical variables.

A two-stage logistic regression approach was employed to identify clinical predictors at ICU admission. In Stage 1 (exploratory), the outcome was “early EN initiation” to identify factors associated with starting EN within 48 h. In Stage 2 (primary), the outcome was “successful early EN advancement” to determine which clinical variables at ICU admission predict not only initiation but also successful advancement—the clinically meaningful endpoint.

For each stage, univariable logistic regression was first performed for all candidate variables, followed by multivariable logistic regression with simultaneous entry of all predictors. Adjusted odds ratios (aORs) with 95% confidence intervals (CIs) were calculated. Model discrimination was assessed using the area under the receiver operating characteristic curve (AUROC), and calibration was evaluated using the Hosmer–Lemeshow goodness-of-fit test. For the Stage 2 model, a preliminary nomogram was developed to support bedside clinical application, and internal validation was performed using bootstrap resampling (B = 200). To examine whether the identified predictors remained consistent across different clinical settings, a sensitivity analysis was performed by repeating both multivariable models separately in medical/neurological and in surgical ICU patients, given that EN practices and clinical priorities often differ substantially between surgical and non-surgical ICU patients.

As this was a retrospective study utilizing all eligible patients during the study period, a formal sample size calculation was not performed. The adequacy of the sample size was assessed using the events per variable (EPV) criterion; with 449 events and 12 predictor variables in the primary analysis, the EPV was 37.4, exceeding the recommended minimum of 10.

*p*-values < 0.05 were considered statistically significant. All analyses were performed using R version 4.0.2 2020 (R Foundation for Statistical Computing, Vienna, Austria).

### 2.5. Ethics Approval and Consent to Participate

This study was approved by the institutional review board of Seoul National University Bundang Hospital (B-2503-960-102; 28 February 2025), and a waiver for written consent was obtained from the institutional review board (IRB).

## 3. Results

### 3.1. Patient Characteristics

**Figure 1:**
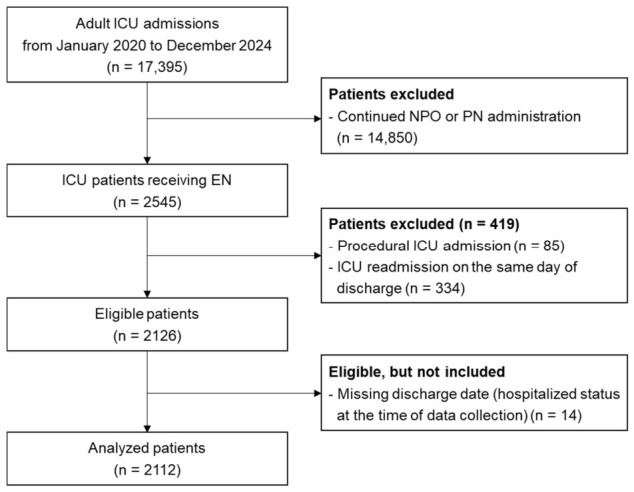
Study enrollment flowchart. EN, enteral nutrition; ICU, intensive care unit; NPO, nothing per oral; PN, parenteral nutrition.

During the 60-month study period from January 2020 to December 2024, there were 17,395 adults admitted to the ICU, of whom 2545 received EN during their ICU stay. After excluding patients with procedural ICU admissions (n = 85) and ICU readmission on the same day of discharge (n = 334), 2126 patients were eligible for analysis. Of those, 14 patients were further excluded due to missing discharge data, resulting in a final analysis cohort of 2112 patients (Figure 1).

The baseline characteristics of the 2112 patients are presented in Table 1 and Table 2, stratified by early EN initiation and successful early EN advancement, respectively. Of the total cohort, 722 patients (34.2%) achieved early EN initiation, of whom 449 (62.2%) subsequently achieved successful early EN advancement, representing 21.3% of the total cohort.

Patients who achieved early EN initiation had significantly lower admission lactate levels (2.6 ± 2.8 vs. 3.0 ± 3.6 mmol/L, *p* = 0.001) and APACHE II scores (27.8 ± 6.5 vs. 28.9 ± 6.7, *p* < 0.001) compared with those who did not. Among the 722 patients who initiated early EN, most baseline characteristics did not differ significantly between those who did and did not subsequently advance, with the exception of vasopressor use, which was more prevalent in the advancement group (64.1% vs. 51.6%, *p* = 0.001).

### 3.2. Association Between Clinical Predictors and Early EN Initiation

Table 3 presents the results of univariable and multivariable logistic regression analysis for early EN initiation. In the multivariable analysis, four predictors were identified. Male sex (aOR 0.84; 95% CI 0.76–0.92; *p* < 0.001), higher admission lactate (aOR 0.84; 95% CI 0.75–0.94; *p* = 0.002), prior surgery (aOR 0.85; 95% CI 0.76–0.96; *p* = 0.008), and higher APACHE II score (aOR 0.86; 95% CI 0.78–0.96; *p* = 0.005) were each significantly associated with a decreased likelihood of achieving early EN initiation.

In the sensitivity analysis restricted to medical/neurological ICU patients (n = 1306), the core predictors remained significant: admission lactate (aOR 0.81; 95% CI 0.70–0.93; *p* = 0.002), APACHE II score (aOR 0.83; 95% CI 0.73–0.94; *p* = 0.003), and male sex (aOR 0.85; 95% CI 0.75–0.95; *p* = 0.004), with effect estimates comparable to those in the primary analysis (Table S1). Additionally, vasopressor use emerged as a significant predictor in this subgroup (aOR 0.86; 95% CI 0.75–0.98; *p* = 0.021). In the surgical ICU subgroup (n = 806), male sex (aOR 0.82; 95% CI 0.70–0.96; *p* = 0.013) and neuromuscular agent use (aOR 0.73; 95% CI 0.58–0.92; *p* = 0.008) were identified as significant negative predictors, while admission lactate and APACHE II score were not significant (Table S2).

### 3.3. Association Between Clinical Predictors and Successful Early EN Advancement

Table 4 presents the univariable and multivariable logistic regression analysis for successful early EN advancement. Male sex (aOR 0.87; 95% CI 0.78–0.96; *p* = 0.008), higher admission lactate (aOR 0.85; 95% CI 0.74–0.96; *p* = 0.009), prior surgery (aOR 0.81; 95% CI 0.70–0.93; *p* = 0.003), and higher APACHE II score (aOR 0.88; 95% CI 0.79–0.99; *p* = 0.037) were each significantly associated with a decreased likelihood of achieving successful early EN advancement.

In the sensitivity analysis restricted to medical/neurological ICU patients (n = 1306), admission lactate (aOR 0.83; 95% CI 0.70–0.97; *p* = 0.022) and APACHE II score (aOR 0.83; 95% CI 0.72–0.96; *p* = 0.011) remained significant predictors, with effect estimates comparable to those in the primary analysis (Table S3). In the surgical ICU subgroup (n = 806), prior surgery was the only significant independent predictor (aOR 0.79; 95% CI 0.65–0.97; *p* = 0.027), while admission lactate and APACHE II score were not significant (Table S4).

### 3.4. Preliminary Nomogram Predicting Successful Early EN Advancement

**Figure 2:**
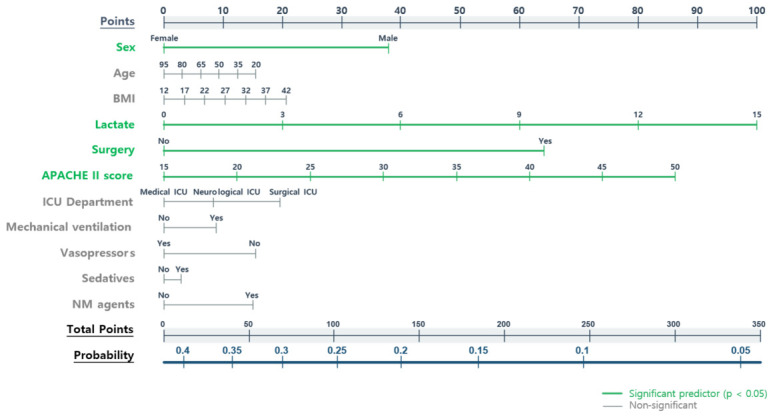
A preliminary nomogram for estimating the probability of successful early EN advancement in critically ill patients. A nomogram derived from the multivariable logistic regression model. Each variable contributes points based on its regression coefficient; higher Total Points indicate a greater burden of adverse predictors and correspond to a lower estimated probability of achieving successful early EN advancement. Statistically significant predictors (male sex, admission lactate, prior surgery, and APACHE II score) are highlighted in green. APACHE II, Acute Physiology and Chronic Health Evaluation II; BMI, body mass index; EN, enteral nutrition; ICU, intensive care unit; NM, neuromuscular.

To illustrate the relative contribution of each predictor, we constructed a preliminary nomogram based on the multivariable logistic regression model for successful early EN advancement (Figure 2). The nomogram integrates male sex, admission lactate level, prior surgery, and APACHE II score to estimate the probability of achieving the outcome. The model demonstrated moderate discrimination (AUROC = 0.598; study prevalence = 21.3%, 449/2112). Practical examples demonstrating the application of the nomogram are provided in Supplementary File S2.

## 4. Discussion

This study evaluated the factors associated with early EN advancement during ICU stay. The significance of our findings is two-fold. First, using clinical variables available at the time of ICU admission, we identified factors significantly associated with both early EN initiation and successful early EN advancement in critically ill patients. Second, we developed a nomogram based on these admission-time variables to provide a practical reference for estimating the probability of successful early EN advancement at the bedside; following external validation, this nomogram may serve as a reference for identifying patients who are less likely to achieve successful early EN advancement.

Admission lactate and APACHE II score were significant predictors at both stages, reflecting that tissue hypoperfusion, metabolic stress, and higher illness severity are fundamental barriers to EN delivery in the ICU [14,17,25]. Elevated admission lactate has been associated with poor perfusion status and gastrointestinal dysmotility that may directly impair tolerance to enteral feeding [26,27]. Male sex and prior surgery were also associated with a lower likelihood of both early EN initiation and successful early EN advancement. The biological basis for this finding may relate to well-established sex differences in critical illness. Male patients tend to exhibit more pronounced metabolic stress responses, stronger hypothalamic–pituitary–adrenal axis activation, and higher rates of vasopressor use and mechanical ventilation—all of which may impair gastrointestinal motility and EN tolerance. Furthermore, androgens have been shown to exert immunosuppressive effects in contrast to the immunoprotective role of estrogens, potentially contributing to worse clinical trajectories in critically ill male patients [28,29]. Surgical patients frequently experience postoperative ileus, hemodynamic instability, and anastomotic concerns that impede early EN [6,30], suggesting that the operative burden itself may be a meaningful barrier to EN delivery regardless of ICU setting.

The sensitivity analysis revealed distinct predictor patterns depending on the ICU setting. When restricted to medical/neurological ICU patients, the analysis confirmed that admission lactate, APACHE II score, and male sex remained significant predictors, supporting the generalizability of the findings in non-surgical contexts. In contrast, the surgical ICU subgroup showed a different pattern—neuromuscular agent use for early EN initiation and prior surgery for successful early EN advancement—while admission lactate and APACHE II score were not significant, suggesting that procedural factors may be more relevant determinants of EN outcomes than systemic disease severity markers in surgical ICU patients.

Our findings carry implications for clinical management, highlighting that a uniform EN approach across ICU types may be insufficient. By utilizing the identified admission-time predictors and the nomogram prototype, early identification of high-risk individuals in medical and neurological ICU patients (based on admission lactate and APACHE II score) may guide the timely initiation of proactive EN strategies. These include closer NST monitoring and earlier escalation to supplemental PN when EN advancement is unlikely. In surgical ICU patients, however, systemic severity markers appear less informative, and EN management may instead benefit from strategies targeting procedural factors—including minimizing unnecessary interruptions during procedures, optimizing postoperative gastrointestinal recovery with prokinetic agents, and developing surgery-specific EN advancement protocols. Taken together, these tailored strategies suggest that ICU-type-specific nutritional care pathways are warranted.

Beyond these subgroup-specific findings, the present study advances the existing literature in several key ways. While previous studies on EN outcomes in critically ill patients have largely focused on feeding intolerance or caloric deficit as single endpoints [23], this study considers a composite outcome capturing both timely initiation and uninterrupted advancement. Although the preliminary predictive nomogram yielded modest discriminative ability given the strict constraint of using only admission-time variables, it provides an invaluable baseline risk assessment before dynamic ICU clinical courses unfold. These findings build upon our previous work demonstrating that rapid EN advancement was associated with reduced in-hospital mortality and shortened hospital stay [18], extending it by identifying who is at risk for failing that goal from the moment of ICU admission.

This study has several limitations. First, the retrospective, single-center design limits causal inference and generalizability. However, the baseline characteristics of our cohort—including age, illness severity, and ICU department distribution—are broadly comparable to those reported in previous multicenter studies of critically ill patients receiving EN [9], suggesting that the findings may have relevance beyond the study institution. Variability in EN practices across ICU types—particularly the lower EN rates observed in surgical ICU patients—may nonetheless reflect differences in postoperative management priorities and represent an acknowledged limitation of this study. Second, detailed surgical subtype data were not individually recorded, precluding direct classification by procedure type. However, as all surgeries performed under general anesthesia were included, their potential influence on EN outcomes may be partially reflected through the prior surgery variable included in the analyses. Third, data on prokinetic agent use were not collected; as these agents may facilitate EN advancement in patients with delayed motility, this represents an unadjusted confounder that should be accounted for in future prospective designs. Lastly, the nomogram presented in this study should be regarded as an initial prototype for admission-time risk estimation rather than a validated clinical decision tool. As it was developed and evaluated within the same cohort, overfitting cannot be excluded. Furthermore, the absence of variables such as surgical subtype and prokinetic agent use may have affected the composition of the multivariable model and the resulting nomogram. Although internal bootstrap validation demonstrated only minimal optimism in the C-statistic, and the model’s discriminative ability was consistent with existing EN-related prediction models in the literature [22], these cannot substitute for external validation. Prospective multicenter studies are warranted to refine and validate the nomogram before clinical implementation.

Despite these limitations, the large sample size, consistency across primary and sensitivity analyses, and the clinical coherence of the identified predictors support the validity of the findings. Identifying high-risk patients for successful early EN advancement at ICU admission—those with male sex, elevated admission lactate, prior surgery, and high APACHE II scores—may allow clinicians to implement proactive nutritional strategies, and we believe that integrating admission-time risk stratification into nutritional care pathways represents a meaningful step toward improving the rate of successful early EN advancement in critically ill patients.

## 5. Conclusions

In conclusion, clinical variables available at the time of ICU admission—specifically male sex, elevated lactate, prior surgery, and higher APACHE II scores—can effectively identify patients at high risk for failing early EN initiation and advancement. Notably, the distinct predictor patterns observed between medical and surgical ICUs underscore the need for ICU-type-specific nutritional pathways rather than a universal protocol. The admission-based nomogram developed in this study provides a pragmatic tool for early risk stratification at the bedside. Future prospective studies are warranted to externally validate this prototype and confirm whether risk-stratified nutritional interventions improve clinical outcomes in critically ill patients.

## Figures and Tables

**Table 1:** Baseline characteristics of the study population stratified by early EN initiation.

Characteristics	Early EN Initiation			
	Overall (n = 2112)	No	Yes	*p*-Value
**Sex, male, n (%)**	1294 (61.3)	893 (69.0)	401 (31.0)	<0.001
**Age, years**	68.3 ± 14.8	68.1 ± 14.7	68.7 ± 15.0	0.414
**BMI, kg/m^2^**	22.5 ± 4.4	22.6 ± 4.2	22.3 ± 4.8	0.095
**Lactate, mmol/L**	2.9 ± 3.3	3.0 ± 3.6	2.6 ± 2.8	0.001
**Surgery, n (%)**				
Yes	558 (26.4)	410 (73.5)	148 (26.5)	<0.001
**APACHE** **II** **score**	28.5 ± 6.7	28.9 ± 6.7	27.8 ± 6.5	<0.001
**ICU department, n (%)**				
Medical ICU	1049 (49.7)	678 (64.6)	371 (35.4)	<0.001
Neurological ICU	257 (12.2)	146 (56.8)	111 (43.2)	
Surgical ICU	806 (38.2)	566 (70.2)	240 (29.8)	
**Mechanical ventilation, n (%)**				
Yes	1192 (56.4)	814 (68.3)	378 (31.7)	0.007
**Administration of medications, n (%)**				
Vasopressors	1376 (65.2)	947 (68.8)	429 (31.2)	<0.001
Sedatives	1026 (48.6)	712 (69.4)	314 (30.6)	0.001
NM agents	1162 (55.0)	818 (70.4)	344 (29.6)	<0.001

Data are presented as n (%) for categorical variables or mean ± SD for continuous variables. APACHE II, Acute Physiology and Chronic Health Evaluation II; BMI, body mass index; EN, enteral nutrition; ICU, intensive care unit; NM, neuromuscular; SD, standard deviation.

**Table 2:** Baseline characteristics of early EN initiators stratified by successful early EN advancement.

Characteristics	Successful Early EN Advancement ^†^			
	Overall (n = 722)	No	Yes	*p*-Value
**Sex, male, n (%)**	401 (55.5)	151 (37.7)	250 (62.3)	0.985
**Age, years**	68.7 ± 15.0	68.0 ± 15.2	69.1 ± 14.8	0.344
**BMI, kg/m^2^**	22.3 ± 4.8	22.1 ± 4.7	22.4 ± 4.9	0.492
**Lactate, mmol/L**	2.6 ± 2.8	2.5 ± 2.6	2.6 ± 2.9	0.851
**Surgery, n (%)**				
Yes	148 (20.5)	64 (43.2)	84 (56.8)	0.152
**APACHE** **II** **score**	27.8 ± 6.5	27.6 ± 7.2	27.9 ± 6.0	0.652
**ICU department, n (%)**				
Medical ICU	371 (51.4)	132 (35.6)	239 (64.4)	0.258
Neurological ICU	111 (15.4)	49 (44.1)	62 (55.9)	
Surgical ICU	240 (33.2)	92 (38.3)	148 (61.7)	
**Mechanical ventilation, n (%)**				
Yes	378 (52.4)	148 (39.2)	230 (60.8)	0.482
**Administration of medications, n (%)**				
Vasopressors	429 (59.4)	141 (32.9)	288 (67.1)	0.001
Sedatives	314 (43.5)	114 (36.3)	200 (63.7)	0.513
NM agents	344 (47.6)	121 (35.2)	223 (64.8)	0.188

^†^ Successful EN advancement is analyzed among patients who achieved early EN initiation (n = 722). Data are presented as n (%) for categorical variables or mean ± SD for continuous variables. APACHE II, Acute Physiology and Chronic Health Evaluation II; BMI, body mass index; EN, enteral nutrition; ICU, intensive care unit; NM, neuromuscular; SD, standard deviation.

**Table 3:** Clinical predictors of early EN initiation: logistic regression analysis.

Characteristics	Unadjusted			Adjusted ^†^		
	OR	95% CI	*p*-Value	OR	95% CI	*p*-Value
**Sex**						
Female	—	—		—	—	
Male	0.84	0.77–0.92	<0.001	0.84	0.76–0.92	<0.001
**Age**	1.04	0.95–1.14	0.412	0.99	0.90–1.01	0.818
**BMI**	0.92	0.84–1.01	0.082	0.94	0.86–1.04	0.225
**Lactate**	0.86	0.78–0.95	0.003	0.84	0.75–0.94	0.002
**Surgery**	0.81	0.74–0.89	<0.001	0.85	0.76–0.96	0.008
**APACHE II score**	0.84	0.77–0.93	<0.001	0.86	0.78–0.96	0.005
**ICU department**						
Medical ICU	—	—		—	—	
Neurological ICU	1.15	1.06–1.23	0.001	1.02	0.93–1.13	0.634
Surgical ICU	0.86	0.78–0.94	0.001	0.91	0.80–1.02	0.114
**Mechanical ventilation**	0.88	0.81–0.97	0.007	1.00	0.91–1.11	0.958
**Administration of medications**						
Vasopressors	0.84	0.76–0.91	<0.001	0.93	0.84–1.04	0.183
Sedatives	0.86	0.78–0.94	0.001	0.98	0.88–1.01	0.700
Neuromuscular agents	0.80	0.73–0.87	<0.001	0.91	0.81–1.03	0.141

^†^ Adjusted for all candidate variables simultaneously. APACHE II, Acute Physiology and Chronic Health Evaluation II; BMI, body mass index; CI, confidence interval; EN, enteral nutrition; ICU, intensive care unit; OR, odds ratio.

**Table 4:** Clinical predictors of successful early EN advancement: logistic regression analysis.

Characteristics	Unadjusted			Adjusted ^†^		
	OR	95% CI	*p*-Value	OR	95% CI	*p*-Value
**Sex**						
Female	—	—		—	—	
Male	0.87	0.78–0.96	0.006	0.87	0.78–0.96	0.008
**Age**	1.07	0.96–1.19	0.210	1.02	0.92–1.15	0.683
**BMI**	0.96	0.87–1.07	0.462	0.98	0.88–1.09	0.668
**Lactate**	0.89	0.79–1.00	0.042	0.85	0.74–0.96	0.009
**Surgery**	0.79	0.70–0.88	<0.001	0.81	0.70–0.93	0.003
**APACHE II score**	0.88	0.80–0.98	0.021	0.88	0.79–0.99	0.037
**ICU department**						
Medical ICU	—	—		—	—	
Neurological ICU	1.06	0.96–1.18	0.233	0.97	0.87–1.09	0.622
Surgical ICU	0.87	0.78–0.97	0.011	0.93	0.81–1.07	0.302
**Mechanical ventilation**	0.88	0.79–0.97	0.012	0.97	0.86–1.09	0.575
**Administration of medications**						
Vasopressors	0.97	0.88–1.08	0.614	1.06	0.94–1.20	0.367
Sedatives	0.90	0.81–1.00	0.054	0.99	0.88–1.12	0.859
Neuromuscular agents	0.87	0.79–0.97	0.010	0.95	0.82–1.09	0.424

^†^ Adjusted for all candidate variables simultaneously. APACHE II, Acute Physiology and Chronic Health Evaluation II; BMI, body mass index; CI, confidence interval; EN, enteral nutrition; ICU, intensive care unit; OR, odds ratio.

## Data Availability

The data presented in this study are available on request from the corresponding author due to the nature of medical records and patient privacy protection regulations, which restrict public sharing of individual-level clinical data.

## Supplementary Materials

The following supporting information can be downloaded at: https://www.mdpi.com/article/10.3390/nu18121977/s1, Supplementary File S1: Sensitivity analysis; Supplementary File S2: Practical examples of nomogram application for predicting successful early enteral nutrition advancement in critically ill patients.
