# Supplementary material for: Beyond Early Initiation: Predictors of Successful Early Enteral Nutrition Advancement in Critically Ill Patients
Source: Nutrients. 2026 Jun 18;18(12):1977. doi: 10.3390/nu18121977 (PMC13305438; doi:10.3390/nu18121977)
Supplement: Supplementary file 1 [file nutrients-18-01977-s001.zip › Supplmentary file S1.pdf]

Supplementary File 1. Sensitivity analysis

Table S1. Sensitivity analysis: clinical predictors of early EN initiation restricted to medical/neurological ICU patients (n = 1,306).

Characteristics	Unadjusted			Adjusted†		
	**OR**	**95% CI**	**p-value**	**OR**	**95% CI**	**p-value**
**Sex**						
Female	—	—		—	—	
Male	0.84	0.75–0.94	0.002	0.85	0.75–0.95	**0.004**
**Age**	1.11	0.99–1.25	0.068	1.08	0.96–1.22	0.226
**BMI**	0.93	0.83–1.04	0.196	0.94	0.84–1.06	0.289
**Lactate**	0.75	0.66–0.86	<0.001	0.81	0.70–0.93	**0.002**
**Surgery**	0.88	0.78–0.99	0.033	0.88	0.78–1.00	0.052
**APACHE II score**	0.79	0.70–0.88	<0.001	0.83	0.73–0.94	**0.003**
**Mechanical ventilation**	0.90	0.80–1.00	0.052	1.01	0.89–1.14	0.905
**Administration of medications**						
Vasopressors	0.78	0.70–0.87	<0.001	0.86	0.75–0.98	**0.021**
Sedatives	0.81	0.72–0.91	<0.001	0.89	0.79–1.02	0.084
Neuromuscular agents	0.83	0.74–0.94	0.001	0.98	0.85–1.13	0.735

†Adjusted for all candidate variables simultaneously. APACHE II, Acute Physiology and Chronic Health Evaluation II; BMI, body mass index; CI, confidence interval; EN, enteral nutrition; ICU, intensive care unit; OR, odds ratio.

Table S2. Sensitivity analysis: clinical predictors of early EN initiation restricted to surgical ICU patients (n = 806).

Characteristics	Unadjusted			Adjusted†		
	OR	95% CI	p-value	OR	95% CI	p-value
**Sex**						
Female	—	—		—	—	
Male	0.83	0.72–0.97	0.017	0.82	0.70–0.96	**0.013**
**Age**	0.86	0.74–0.99	0.041	0.87	0.74–1.02	0.080
**BMI**	0.94	0.80–1.10	0.414	0.96	0.82–1.13	0.644
**Lactate**	1.04	0.89–1.20	0.649	1.00	0.86–1.17	0.980
**Surgery**	0.84	0.72–0.97	0.020	0.91	0.76–1.08	0.277
**APACHE II score**	0.89	0.76–1.04	0.129	0.92	0.78–1.09	0.355
**Mechanical ventilation**	0.88	0.76–1.03	0.104	0.98	0.82–1.16	0.797
**Administration of medications**						
Vasopressors	0.97	0.84–1.13	0.709	1.08	0.90–1.31	0.428
Sedatives	1.03	0.88–1.19	0.747	1.22	1.00–1.49	0.057
Neuromuscular agents	0.82	0.71–0.95	0.010	0.73	0.58–0.92	**0.008**

†Adjusted for all candidate variables simultaneously. APACHE II, Acute Physiology and Chronic Health Evaluation II; BMI, body mass index; CI, confidence interval; EN, enteral nutrition; ICU, intensive care unit; OR, odds ratio.

Table S3. Sensitivity analysis: clinical predictors of successful early EN advancement restricted to medical/neurological ICU patients (n = 1,306).

**Characteristics**	**Unadjusted**			**Adjusted**†		
	**OR**	**95% CI**	**p-value**	**OR**	**95% CI**	**p-value**
**Sex**						
Female	—	—		—	—	
Male	0.88	0.77–1.00	0.047	0.89	0.78–1.02	0.082
**Age**	1.13	0.99–1.29	0.072	1.11	0.96–1.27	0.157
**BMI**	0.96	0.85–1.10	0.556	0.97	0.85–1.11	0.695
**Lactate**	0.80	0.68–0.93	0.004	0.83	0.70–0.97	**0.022**
**Surgery**	0.93	0.81–1.07	0.309	0.94	0.82–1.09	0.429
**APACHE II score**	0.82	0.72–0.94	0.003	0.83	0.72–0.96	**0.011**
**Mechanical ventilation**	0.94	0.83–1.07	0.362	1.04	0.90–1.20	0.618
**Administration of medications**						
Vasopressors	0.92	0.81–1.05	0.212	1.00	0.87–1.16	0.983
Sedatives	0.85	0.74–0.97	0.013	0.88	0.77–1.02	0.095
Neuromuscular agents	0.91	0.80–1.04	0.161	1.00	0.85–1.17	0.958

†Adjusted for all candidate variables simultaneously. APACHE II, Acute Physiology and Chronic Health Evaluation II; BMI, body mass index; CI, confidence interval; EN, enteral nutrition; ICU, intensive care unit; OR, odds ratio.

Table S4. Sensitivity analysis: clinical predictors of successful early EN advancement restricted to surgical ICU patients (n = 806).

Characteristics	Unadjusted			Adjusted†		
	**OR**	**95% CI**	**p-value**	**OR**	**95% CI**	**p-value**
**Sex**						
Female	—	—		—	—	
Male	0.84	0.71–1.00	0.050	0.84	0.70–1.01	0.068
**Age**	0.91	0.76–1.08	0.275	0.91	0.76–1.10	0.330
**BMI**	0.99	0.82–1.18	0.876	0.99	0.82–1.20	0.933
**Lactate**	1.02	0.86–1.22	0.798	0.96	0.79–1.16	0.657
**Surgery**	0.74	0.62–0.88	<0.001	0.79	0.65–0.97	**0.027**
**APACHE II score**	0.96	0.80–1.15	0.657	1.00	0.82–1.22	0.993
**Mechanical ventilation**	0.78	0.66–0.93	0.005	0.86	0.70–1.05	0.144
**Administration of medications**						
Vasopressors	1.12	0.93–1.34	0.235	1.22	0.97–1.54	0.090
Sedatives	1.11	0.92–1.33	0.270	1.27	1.00–1.62	0.052
Neuromuscular agents	0.88	0.74–1.04	0.138	0.77	0.59–1.00	0.050

†Adjusted for all candidate variables simultaneously. APACHE II, Acute Physiology and Chronic Health Evaluation II; BMI, body mass index; CI, confidence interval; EN, enteral nutrition; ICU, intensive care unit; OR, odds ratio.
