# Supplementary material for: Beyond Early Initiation: Predictors of Successful Early Enteral Nutrition Advancement in Critically Ill Patients
Source: Nutrients. 2026 Jun 18;18(12):1977. doi: 10.3390/nu18121977 (PMC13305438; doi:10.3390/nu18121977)
Supplement: Supplementary file 1 [file nutrients-18-01977-s001.zip › Supplmentary file S2.pdf]

Supplementary File 2. Practical examples of nomogram application for predicting successful early enteral nutrition advancement in critically ill patients

How to use the nomogram

The nomogram (Figure 2) allows bedside estimation of the probability of successful early EN advancement using clinical variables available at ICU admission. Each variable has a corresponding axis, and the individual points are summed to obtain a Total Points value, which is then mapped to an estimated probability. In this nomogram, higher Total Points indicate a greater burden of adverse predictors and correspond to a lower probability of successful early EN advancement.

Step 1. Locate each predictor variable on its corresponding row in the nomogram (Figure 2).
Step 2. Draw a vertical line upward from the patient's value to the Points axis (top row) and read the corresponding point value for each variable.
Step 3. Sum the individual points from all variables to obtain the Total Points.
Step 4. Locate the Total Points on the Total Points axis and draw a vertical line downward to the Probability axis. Higher Total Points correspond to a lower estimated probability of successful early EN advancement.

Note: Statistically significant predictors (male sex, admission lactate, prior surgery, and APACHE II score) are marked with (★) and highlighted in the tables below. Point values are approximate and should be read directly from Figure 2 of the manuscript. For each significant predictor, the adverse value (e.g., male sex, higher lactate, surgery yes, higher APACHE II) yields higher points, reflecting increased risk of EN advancement failure.

<u>Case 1. High-risk patient: low probability of successful early EN advancement</u>

A 70-year-old male (20 kg/m² BMI) and a history of underlying cardiovascular disease presented with an acute abdomen. He underwent an emergency exploratory laparotomy, which revealed acute mesenteric ischemia complicated by focal bowel perforation. Postoperatively, the patient was admitted to the SICU in a state of severe septic shock and acute respiratory distress syndrome (ARDS). Upon ICU admission, he was intubated and maintained on invasive mechanical ventilation. To achieve hemodynamic stability, continuous infusions of high-dose vasopressors (norepinephrine) were required, alongside deep sedation; however, neuromuscular blocking agents were not administered during the initial admission period. Baseline laboratory workup at the time of admission indicated profound systemic tissue hypoperfusion, characterized by a markedly elevated admission lactate of 10.0 mmol/L and a high overall baseline APACHE II score of 38.

Variables	Value	Points
Sex ★	Male	38
Age (years)	70	5
BMI (kg/m²)	20	6
Admission lactate (mmol/L) ★	10.0	67
Prior surgery ★	Yes	64
APACHE II score ★	38	57
ICU department	Surgical ICU	20
Mechanical ventilation	Yes	9
Vasopressors	Yes	0
Sedatives	Yes	3
Neuromuscular Blocking Agents	No	0
**Total Points**		~ 268
**Estimated probability of successful early EN advancement**		~ 8.6%

Clinical Interpretation

This patient accumulates a high Total Points (~268) due to multiple adverse predictors: male sex (38 pts), markedly elevated lactate of 10 mmol/L (67 pts), prior surgery (64 pts), and high APACHE II score of 38 (57 pts). The high Total Points corresponds to an estimated probability of only ~8.6% for achieving successful early EN advancement, indicating a very high risk of EN failure. Clinicians should consider proactive nutritional strategies including closer monitoring of EN tolerance, protocol-driven advancement, and early evaluation of supplemental parenteral nutrition.

Case 2. Lower-risk patient: higher probability of successful early EN advancement

A 45-year-old female (18 kg/m$^{2)}$ BMI and no significant prior medical history was admitted to the MICU due to community-acquired pneumonia complicated by acute respiratory distress. Upon admission, her respiratory status was managed effectively with high-flow nasal cannula (HFNC) oxygen therapy, without requiring invasive mechanical ventilation. Clinically, the patient remained hemodynamically stable on minimal vasopressor support (norepinephrine at 0.01 mcg/kg/min) and did not require the administration of any sedatives or neuromuscular blocking agents. She had no recent history of surgical intervention. Baseline laboratory workup at the time of ICU admission revealed a mild systemic stress response, characterized by an admission lactate of 2.0 mmol/L and a baseline APACHE II score of 16.

Variables	Value	Points
Sex ★	Female	0
Age (years)	45	10
BMI (kg/m^2)	18	5
Admission lactate (mmol/L) ★	2.0	13
Prior surgery ★	No	0
APACHE II score ★	16	2
ICU department	Medical ICU	0
Mechanical ventilation	No	0
Vasopressors	Yes	0
Sedatives	No	0
Neuromuscular Blocking Agents	No	0
**Total Points**		~ 31
**Estimated probability of successful early EN advancement**		~ 37%

Clinical Interpretation

This patient accumulates low Total Points (~31) due to favorable predictors across all significant variables. The low Total Points corresponds to an estimated probability of approximately ~37% for achieving successful early EN advancement, considerably higher than Case 1. Standard EN initiation and advancement protocols are appropriate, with routine monitoring.
